# Contribution of genetic test results to patient management in ophthalmology: results from a Turkish Stargardt disease cohort

**DOI:** 10.55730/1300-0144.6077

**Published:** 2025-08-19

**Authors:** Fulya YAYLACIOĞLU TUNCAY, Şengül ÖZDEK, Burak ACAR, Gülsüm KAYHAN, Murat YÜKSEL, Hüseyin Baran ÖZDEMİR, Gökhan GÜRELİK, Mehmet Ali ERGÜN

**Affiliations:** 1 Department of Medical Biology, Gülhane Faculty of Medicine, University of Health Sciences, Ankara, Turkiye; 2 Department of Ophthalmology, Faculty of Medicine, Gazi University, Ankara, Turkiye; 3 Department of Medical Genetics, Faculty of Medicine, Gazi University, Ankara, Turkiye

**Keywords:** Stargardt disease, *ABCA4*, genetic testing, multidisciplinary approach, inherited eye diseases

## Abstract

**Background/aim:**

The aim of the study was to analyze the genotype and phenotype characteristics of Turkish patients with a clinical diagnosis of Stargardt disease and to evaluate how collaboration between the departments of medical genetics and ophthalmology contributes to patient management.

**Materials and methods:**

The clinical findings, genetic testing workflow in the medical genetics department, and the genetic testing results of patients clinically diagnosed with Stargardt disease in the ophthalmology department were retrospectively analyzed.

**Results:**

The study included 50 patients from 46 families. The genetic test reports confirmed the clinical diagnosis of Stargardt disease type 1 (STGD1) in 27 patients (54%), led to revision of the clinical diagnosis in five patients (10%), and were inconclusive in 18 patients (36%). A total of 26 pathogenic *ABCA4* variants were reported in 39 patients, three of which were novel: c.466_467dupAT, p.Leu157SerTer2; c.4540-1G>C; c.878delC, p.Met293SerfsTer7. The most recurrent *ABCA4* variant was c.5882G>A, p.Gly1961Glu detected in 10 unrelated patients. Patients with biallelic severe *ABCA4* variants or biallelic loss of function variants had an earlier age of ascertainment (p = 0.024 and p = 0.008, respectively). The mean interval between the referral of patients from the ophthalmology clinic and the first visit with the medical geneticist was 13.8 days, and the mean time to receive genetic test results with posttest counseling was 6.9 months after the first visit.

**Conclusion:**

This study serves as a representative example of how genetic testing and a multidisciplinary approach can contribute to management of inherited eye diseases. It also reports three novel *ABCA4* variants in Turkish patients with Stargardt disease and describes genotype–phenotype correlations. However, conducting multicenter studies with larger sample sizes from Türkiye will be essential to broaden the spectrum of *ABCA4* variants and enhance our understanding of genotype–phenotype relationships.

## 1. Introduction

Stargardt disease type 1 (STGD1, OMIM 248200) is the most common form of inherited macular dystrophy in children and young adults [1]. The inheritance pattern is autosomal recessive (AR), and biallelic disease-associated variants in the ATP-binding cassette subfamily A member 4 (*ABCA4*) gene cause functional and structural retinal damage, resulting in STGD1 [2]. The diagnosis of STGD1 has been mainly based on phenotypic characteristics since the first description of the disease. However, STGD1 exhibits great clinical variability, and clinical diagnosis can be confirmed genetically using available molecular diagnostic tests.

The *ABCA4* gene is located on chromosome 1p21–p22, consists of 50 exons, and encodes a large protein of 2273 amino acids that belongs to the ATP-binding cassette transporter family [2]. ABCA4 is expressed both in cones and rods [3, 4]. The defective function of this protein results in the accumulation of N-retinylidene-N-retinylethanolamine (A2-E), the main component of lipofuscin, in the retinal pigment epithelium (RPE). The increased lipofuscin in RPE cells causes oxidative damage and promotes cell death [5]. Loss of the RPE layer results in secondary photoreceptor death, and retinal degeneration progresses [6]. *ABCA4* shows great allelic heterogeneity, with more than 2800 variants reported to date [7]. The majority of *ABCA4* variants are missense. However, protein-truncating variants including frameshift, nonsense, and splicing variants were reported in patients. Intronic variants and complex alleles with more than one variant have also been described for the *ABCA4* gene [8, 9]. The extensive allelic heterogeneity in this gene coincides with a broad phenotypic spectrum, ranging from progressive cone-rod dystrophy (CORD) with early onset to mild maculopathy with late onset or even an asymptomatic course [10]. Therefore, the term ABCA4 retinopathies (ABCA4R) has been introduced to describe a wide range of phenotypes, including STGD1, fundus flavimaculatus, bull’s eye maculopathy, CORD, and retinitis pigmentosa (RP) [11]. The residual function of the ABCA4 protein, defined by variant characteristics, correlates with disease severity in ABCA4Rs [7, 12]. Therefore, the effects of *ABCA4* variants on protein function, as well as their composition in individual patients, define the clinical manifestations and disease severity.

As the main component of ABCA4R, STGD1 commonly presents in childhood with progressive bilateral central vision loss, although it is a highly heterogeneous disease regarding genotype and phenotype. The clinical diagnosis of STGD1 is further complicated by phenotypic overlaps with Malattia Levantinese, pattern macular dystrophy, enhanced S-cone syndrome, achromatopsia, Stargardt-like diseases (STGD3 and STGD4), and, in severe cases, CORD and RP. Additionally, late-onset STGD1 is mostly misdiagnosed with age-related macular degeneration (AMD) [13]. Therefore, a multidisciplinary approach involving medical geneticists gains importance for reaching a molecular diagnosis to eliminate clinical uncertainty and assist in prognostication [7, 14, 15], estimate recurrence risk [7, 16], and provide a basis for gene-specific treatments [17]. In addition, such an approach may identify new gene variants, clarify their relationship with clinical findings, and expand the genotype–phenotype spectrum.

The distribution and frequency of disease-associated variants may differ across regional, racial, and ethnic groups in inherited diseases, and there are few reports evaluating the clinical and/or genetic characteristics of patients diagnosed with Stargardt disease in Türkiye [18, 19]. Moreover, to our knowledge, there are no reports analyzing the process of genetic testing and its contribution to patient management in ophthalmology clinics. Therefore, in this study, we first aimed to evaluate the genotype and phenotype characteristics of Turkish patients clinically diagnosed with Stargardt disease. Second, we aimed to provide information on how collaboration between the departments of medical genetics and ophthalmology contributes to patient management, using Türkiye as a representative example of a country without specialized clinics for inherited eye diseases.

## 2. Materials and methods

### 2.1. Study design and ethical approval

This retrospective study was designed to evaluate the genetic test results of patients with a clinical diagnosis of Stargardt disease. Medical records, imaging studies, and genetic test reports were retrospectively reviewed. The research protocol was approved by the Gazi University Institutional Ethical Review Board (approval no: 2023–751599) and the study was conducted in compliance with the Declaration of Helsinki. Written informed consent was obtained from all participants and/or their legal guardians.

### 2.2. Participants

Patients with a clinical diagnosis of Stargardt disease, followed in the Department of Ophthalmology at Gazi University Faculty of Medicine and referred to the Department of Medical Genetics between January 2019 and December 2023, were included.

### 2.3. Clinical evaluation

**Figure 1:**
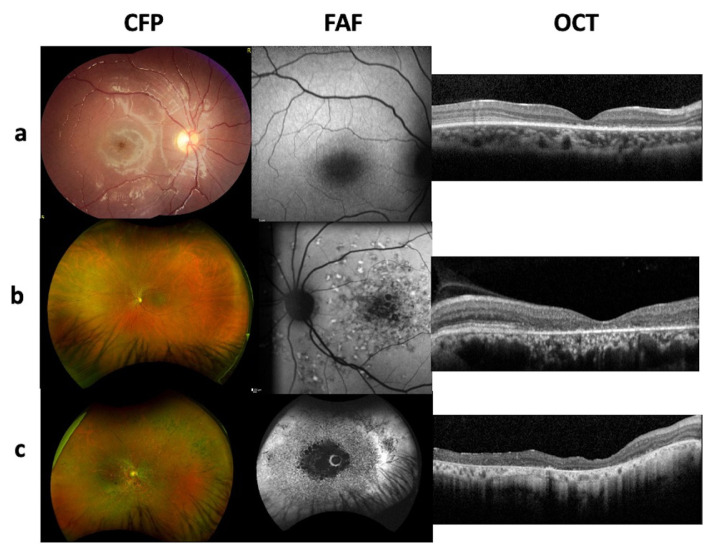
Color fundus photography (CFP), fundus autofluorescence (FAF), and optical coherence tomography (OCT) images of patients with Stargardt disease. (a) Patient 7 with the type I phenotype: central outer retinal atrophy on OCT with no hypoautofluorescence outside the macula. (b) Patient 10 with the type II phenotype: outer retinal atrophy throughout the scanned area with peripapillary sparing on OCT, homogenous hypoautofluorescence in the macula, and heterogenous hypoautofluorescence and hyperautofluorescence extending to the arcuate vessels. (c) Patient 5 with the type III phenotype: outer retinal atrophy throughout the scanned area on OCT, homogenous hypoautofluorescence between the arcuate vessels, and heterogenous hypoautofluorescence and hyperautofluorescence extending to the midperiphery.

All patients underwent comprehensive ophthalmological examinations comprising best-corrected visual acuity (BCVA), assessed with the Snellen chart and categorized into six groups for phenotypic comparison (Group 0: ≥ 0.8 to Group 5: no light perception) [20]; biomicroscopy; color fundus photography (CFP); fundus autofluorescence imaging (FAF; Heidelberg Engineering Inc., Heidelberg, Germany); and optical coherence tomography (OCT; Heidelberg Engineering Inc., Heidelberg, Germany). A retina specialist made the clinical diagnosis of Stargardt disease based on patients’ visual symptoms, ophthalmological examinations, and findings from OCT and FAF images. Electrophysiological testing could not be included in this study due to unavailability in all patients. Patients were further classified according to FAF findings using the system of Fujinami et al. [21], which defines three types. Type I is characterized by an area of foveal hypoautofluorescence surrounded by a retina with a homogeneous appearance, with or without foci of high or low autofluorescent signals (Figure 1a). Type II is characterized by an area of foveal hypoautofluorescence surrounded by a heterogeneous region containing hyperautofluorescent and hypoautofluorescent points that extend to the temporal arcades, giving the retina a reticulate appearance, while the peripheral retina remains normal (Figure 1b). Type III is characterized by extensive areas of hypoautofluorescence in the posterior pole and a heterogeneous appearance of the remaining retinal areas, with both hyperautofluorescent and hypoautofluorescent points (Figure 1c). The presence or absence of flecks on CFP and FAF images was also recorded for genotype–phenotype correlation.

### 2.4. Genetic analysis

Genomic DNA was extracted from peripheral venous blood using the QIAamp DNA mini kit (Qiagen, Hilden, Germany). The clinical exome sequencing panels CES_v2 and CES_v3 (Sophia Genetics SA, Saint-Sulpice, Switzerland) were used for library preparation and exome enrichment. All procedures were carried out according to the manufacturer’s protocols. Paired-end sequencing was performed on an Illumina NextSeq 500 system (Illumina Inc., San Diego, CA, USA) with a read length of 2′ 150 bp. All bioinformatics analyses were performed on the Sophia DDM platform (Sophia Genetics SA, Saint-Sulpice, Switzerland), which includes algorithms for alignment; calling SNPs and small indels (Pepper); detecting copy number variations (Muskat); and functional annotation (Moka). Raw reads were aligned to the human reference genome (GRCh37/hg19). Variant filtering was performed on the Sophia DDM platform (Sophia Genetics SA, Saint-Sulpice, Switzerland). All variants were classified according to the standards of the American College of Medical Genetics and Genomics (ACMG) and the Association for Clinical Genomic Science (ACGS) [22, 23].

For genotype–phenotype comparisons, the variants were classified as mild, moderate, or severe based on the criteria proposed by Lee et al. [24] and the classification table of Bianco et al. [14], as presented in Table 1. These classifications are based on genotype–phenotype correlation analyses derived from the long-term prognostic outcomes of 112 and 71 genetically confirmed patients with ABCA4-associated disease, respectively [14, 24].

The study also analyzed the percentage of conclusive genetic test results; the duration between referral from the ophthalmology clinic and the first visit with a medical geneticist; the time required to receive test results with posttest genetic counseling after the first visit; and the percentage of patients who received genetic counseling before and after testing for future pregnancies, prognosis, or inconclusive results. These metrics were used to evaluate the clinical utility of genetic testing in ophthalmology for patient management.

### 2.5. Statistical analysis

All graphs, calculations, and statistical analyses were performed using GraphPad Prism version 8.0 for Mac (GraphPad Software, San Diego, CA, USA). Categorical variables were compared using the Pearson chi-square and Fisher exact tests. A p value less than 0.05 was considered significant.

## 3. Results

This study included 50 patients from 46 families, comprising three sibling groups. All patients were clinically diagnosed with Stargardt disease by a retina specialist and subsequently underwent genetic testing by medical geneticists.

Tables 1–3 present the patients’ genetic test results and clinical findings. The cohort comprised individuals from 27 different provinces across all seven geographical regions of Türkiye (Supplementary Table 1). The participants all self-reported as being Turkish. The mean age at examination was 25.77 years (range: 6–69 years). A total of 21 participants (42%) were male, and 29 (58%) were female. Parental consanguinity was reported in 34 patients (68%). Vision loss was the most frequently reported initial symptom (84%). The patient-reported age of onset (age of ascertainment, AOA) was 15.84 years (range: 1–62 years). BCVA at examination ranged from counting fingers (CF) at 1 m to 1.0. Fundus examination revealed macular atrophy, with or without flecks, in most patients (68%). Additional findings included widespread retinal atrophy with or without pigmentary changes, paracentral atrophy with foveal sparing, retinal exudates, beaten bronze appearance, bull’s eye maculopathy, and subtle macular changes. FAF and OCT imaging were available for all patients, with central hypoautofluorescence and central outer retinal atrophy being the most frequently observed features (Table 1–3, and Supplementary Table 1).

Genetic test results were conclusive in 32 patients. Among these, 27 patients (54%) had genotypes consistent with a clinical diagnosis of STGD1, exhibiting biallelic pathogenic (P) or likely pathogenic (LP) variants in the *ABCA4* gene (Table 1). In five patients, the genetic findings prompted reevaluation of the clinical diagnosis, identifying pathogenic or likely pathogenic variants in other genes associated with different retinal phenotypes, including achromatopsia, enhanced S-cone syndrome, and cone-rod dystrophy (CORD) (Table 2). The genetic test results were inconclusive for the remaining 18 patients (36%). Of these patients, seven had monoallelic pathogenic variants in *ABCA4*, one had biallelic variants of uncertain significance (VUS) in *ABCA4*, two had monoallelic VUS in *ABCA4*, and eight harbored VUS in other retinal dystrophy-related genes, including *KCNV2, RIMS1, CRB1, CFH, RP1L1, UNC119*, and *SEMA4A* (Table 3).

### 3.1. *ABCA4* variants

A total of 36 different *ABCA4* variants (26 P/LP and 10 VUS) were reported in 39 patients (Tables 1 and 3). Among pathogenic *ABCA4* variants, 12 were missense, five were nonsense, four were frameshift, three were splicing, and two were intronic (Supplementary Table 2). The variants detected in this study are distributed throughout the *ABCA4* gene, including both exonic and intronic regions (Supplementary Table 2).

Of the 39 patients, 13 had homozygous pathogenic variants, 14 had compound heterozygous variants, and five had monoallelic pathogenic variants. The remaining seven patients carried biallelic or monoallelic VUS in *ABCA4* (Supplementary Table 2). Complex alleles were detected in seven patients (Supplementary Table 2).

In this study, the most recurrent *ABCA4* variant was c.5882G>A, p.Gly1961Glu, detected in 10 unrelated patients and homozygous in one of them (P6). The c.4793C>A (p.Ala1598Asp) variant was the second most common, detected in six patients from four families, and was homozygous in one family including P7, P8, and P9. Additionally, five different variants were detected in more than one family in the study population (Supplementary Table 2).

Among the 26 pathogenic *ABCA4* variants, three were reported as novel in this study: c.466_467dupAT (p.Leu157SerTer2); c.4540-1G>C; and c.878delG (p.Met293SerfsTer7) (Table 1). Among these novel variants, c.466_467dupAT and c.4540-1G>C were homozygous and classified as pathogenic (PVS1, PM2, PM3_P, PP4) and likely pathogenic (PVS1_M, PM2, PM3_P, PP4), respectively. The c.878delG variant was compound heterozygous with c.5882G>A, p.Gly1961Glu, and was classified as likely pathogenic (PVS1, PM2, PM3, PP4).

### 3.2. Genotype–phenotype relationships in patients with conclusive genetic test results

**Figure 2:**
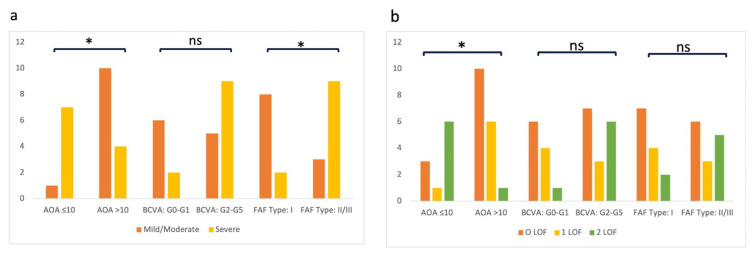
Graphs showing the phenotype spectrum of patients with conclusive genetic test results for STGD1. (a) Comparison of patients with different *ABCA4* variant severities (severe versus mild/moderate). (b) Comparison of patients with different numbers of loss-of-function (LOF) variants in the *ABCA4* gene (0 LOF, 1 LOF, 2 LOF). The x-axis indicates the number of patients in different groups: AOA (≤ 10 years, > 10 years), BCVA groups (G0–G1, G2–G5), and FAF types (type I and type II–III). ns, not significant (p > 0.05); *, statistically significant (p < 0.05).

The phenotypic characteristics were compared in 27 patients harboring biallelic disease-associated *ABCA4* variants (Figure 2). The severity of these variants is listed in Table 1. Patients carrying biallelic severe *ABCA4* variants exhibited more severe FAF findings (p = 0.010) and an earlier AOA (p = 0.024), as illustrated in Figure 2a. Other phenotypic features, including BCVA groups and the presence of flecks, did not differ significantly between genotype groups (p > 0.05). Patients were further categorized into three groups based on the number of *ABCA4* alleles with a loss-of-function (LOF) variant, including nonsense, frameshift, and splicing variants (Supplementary Table 2). Patients with two LOF variants demonstrated an earlier AOA (p = 0.008), as shown in Figure 2b. However, BCVA or FAF types did not differ significantly between LOF groups (p > 0.05). When the presence of flecks was compared between phenotype and genotype groups, patients with flecks exhibited more severe FAF findings (p = 0.018). However, the presence of flecks did not differ across other phenotypic characteristics or genotype groups (p > 0.05). Notably, patients with an AOA greater than 10 years and type I FAF findings lacked biallelic severe or LOF variants (Table 1; Supplementary Table 1).

In this cohort, eight patients carried the c.5882G>A (p.Gly1961Glu) variant in *ABCA4*, previously reported as mild. None of the patients with this variant had type III FAF findings or BCVA worse than 0.1 (Table 1). The mean AOA was 23.4 (range: 14–45 years) for patients with this variant. P4, homozygous for the p.Gly1961Glu variant, presented with a mild phenotype (Table 1). P20, carrying an additional frameshift variant on the second allele, exhibited type II FAF findings at age 61, with a BCVA of 0.2 (Table 1).

This cohort included two families with biallelic *ABCA4* variants. P2 and P3 were siblings with a homozygous frameshift variant (c.5917delG, p.Val1973Ter). P9, P10, and P11 were siblings with a homozygous missense variant (c.4793C>A, p.Ala1598Asp). Phenotypic findings were similar among family members, as shown in Table 1.

Two novel *ABCA4* variants, p.Leu157SerTer2 and c.4540-1G>C, were identified in a homozygous state in P7 and P14, respectively. For both variants, the AOA was below 10 years, and the FAF was type I at the time of examination (6 years of age). Another novel *ABCA4* variant, p.Met293SerfsTer7, was identified together with a mild variant, p.Gly1961Glu, in P24. For this patient, the AOA was reported as 20 years, and the FAF was type I at 40 years of age. For three patients, the fundus findings did not involve flecks.

**Figure 3:**
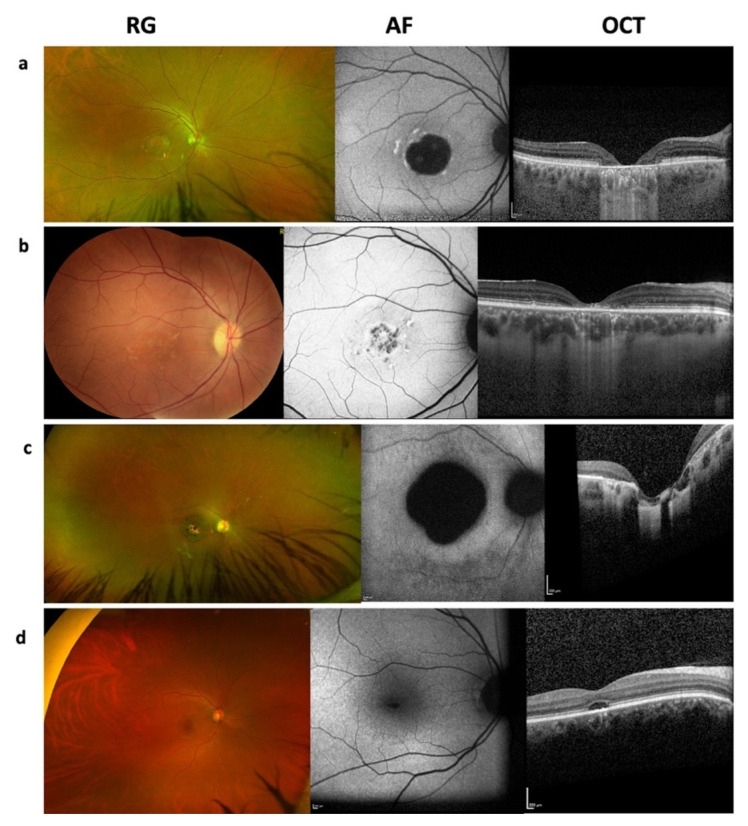
Differential diagnoses in patients with clinical suspicion of Stargardt disease. Color fundus photography (CFP), fundus autofluorescence (FAF), and optical coherence tomography (OCT) images. (a) Patient 23: late-onset STGD1. (b) Patient 31: monoallelic *ABCA4* variant. (c) Patient 44: cone-rod dystrophy 12. (d) Patient 46: achromatopsia.

Late-onset STGD1, defined as an age of onset of 45 years or older, was clinically suspected in three patients in our cohort: P23, P30, and P37 (Tables 1 and 3). The AOA was 45 years in P23, who had milder clinical findings with central macular atrophy with flecks, type I FAF findings, and central outer retinal atrophy on OCT (Figure 3a). The clinical diagnosis of Stargardt disease in P23 was supported by genetic testing, which revealed one mild and one severe *ABCA4* allele (Table 1). However, genetic testing results were inconclusive in P30, who carried a monoallelic pathogenic *ABCA4* variant, and in P37, who carried a monoallelic VUS in *ABCA4* and biallelic VUS in *CFH* (Table 3; Supplementary Table 1). Therefore, clinical and genetic reevaluation, as well as follow-up, were planned for these patients.

Monoallelic pathogenic *ABCA4* variants were detected in seven patients in this cohort (Table 3). Patients with monoallelic pathogenic variants mostly showed central or paracentral atrophy without flecks except P30 and P31 (Figure 3b).

In this cohort, five patients clinically suspected of having Stargardt disease received a genetic diagnosis of different retinal dystrophies (Table 2). P38 carried biallelic pathogenic *CNGA3* variants associated with autosomal recessive achromatopsia 2. P46 carried biallelic pathogenic *CNGB3* variants associated with autosomal recessive achromatopsia 3. Both patients presented with early-onset symptoms, including nystagmus and strabismus, and central atrophy was detected without flecks on fundus examination. P46 also showed a hyporeflective optical gap appearance in OCT (Figure 3d). P39 carried a monoallelic pathogenic *GUCY2D* variant associated with autosomal dominant CORD. This patient also carried a monoallelic p.Gly1961Glu variant in *ABCA4* together with two intronic variants of uncertain significance in the cis position. Central vision loss began in early childhood, and fundus examination revealed subtle changes with an optical gap appearance on OCT. During the reevaluation process, full-field electroretinography (ERG) was performed, revealing decreased cone and rod responses, with cone responses more severely affected than rods. P44 carried homozygous pathogenic variants in *PROM1* associated with autosomal recessive CORD. Fundus findings included central retinal atrophy without flecks, and BCVA was counting fingers at 1 m at 28 years of age (Figure 3c). P48 carried homozygous *NR2E3* variants associated with enhanced-S-cone syndrome. P48 was 8 years old, presenting with decreased vision. However, after the genetic diagnosis, his parents were asked again about night vision problems, and they admitted that his vision was worse at night. Central retinal atrophy with fibrotic scars sparing the fovea and fleck-like lesions were observed in this patient. Full-field ERG performed during reevaluation showed absent rod responses on scotopic testing and low-amplitude, delayed 30 Hz flicker responses (Table 2).

### 3.3. Contribution of genetic test results to the clinical evaluation

After clinical evaluation by an ophthalmologist, all patients included in the study were referred to a medical geneticist for genetic testing. The medical geneticist provided each patient with both pretest and posttest genetic counseling. The mean duration between referral from the ophthalmology clinic and the first visit with the medical geneticist was 13.8 days (range: 1–62 days; Supplementary Table 1). The mean duration from the first visit with the geneticist to receipt of the genetic test results, including the posttest counseling session, was 6.9 months (range: 4–13 months; Supplementary Table 1).

Genetic test results confirmed the clinical diagnosis of Stargardt disease (STGD1) in 54% of patients, while in 10% of cases the clinical diagnosis was revised. A total of 10 families were informed about the disease risk for future pregnancies and the opportunities for prenatal genetic diagnosis, as they already had one affected child with STGD1. All patients with confirmed genetic diagnoses were counseled by their ophthalmologists regarding prognosis in light of their clinical findings and the identified disease-associated *ABCA4* variants. The medical geneticist informed patients with inconclusive test results about their follow-up. Segregation analysis and examination of other family members were planned for P35, who carried a homozygous VUS in *ABCA4* and a heterozygous VUS in *PROM1*.

## 4. Discussion

In this study, the largest single-center cohort of Turkish patients with a clinical diagnosis of Stargardt disease was analyzed in terms of genotype and phenotype characteristics. There were only three previous reports evaluating both clinical and genetic properties of Turkish patients with Stargardt disease. In 2004, Özgül et al. evaluated all exons of *ABCA4* in five patients with Stargardt disease and 35 patients with autosomal recessive RP [18]. In 2016, Bardak et al. evaluated *ELOVL4* and *PRPH2* genes in 30 Turkish patients with Stargardt disease [25]. In 2024, Sinim Kahraman et al. reported 30 *ABCA4* variants in 27 Turkish patients with STGD1 [19].

In our study cohort, 54% of patients had conclusive genetic test results confirming the diagnosis of STGD1. Additionally, 10% of patients had conclusive genetic test results confirming other retinal dystrophies listed in the differential diagnosis of Stargardt disease. The remaining 36% had inconclusive genetic test results. In Türkiye, there have been no reports evaluating the percentage of conclusive genetic test results in Stargardt disease or in other inherited retinal dystrophies (IRD) using next-generation sequencing. The rate of conclusive molecular diagnosis was reported as 50% to 60% in several studies like our study [26–28]. However, a study from Brazil reported this rate to be as high as 80% using a gene panel for macular dystrophies [29]. Another study from Spain reported this rate to be 75% with exome sequencing and screening of *ABCA4* introns [30].

The inconclusive cases in our cohort included patients with monoallelic pathogenic *ABCA4* variants (5/18), monoallelic or biallelic VUS in *ABCA4* (7/18), or VUS in other retinal dystrophy genes (6/18). Zernant et al. showed that 20%–25% of Stargardt disease patients were monoallelic and 15% had no identified *ABCA4* variant [31]. The inconclusive cases in our cohort could be explained by undetected deep intronic or regulatory variants and structural rearrangements. In addition, hypomorphic alleles, variants incorrectly classified as benign due to their relatively high carrier frequency, or phenocopies could account for inconclusive cases [32]. Variants in genes not yet associated with the disease may also contribute. To resolve such cases, follow-up strategies may include targeted copy number variant (CNV) analysis; whole-genome sequencing with structural variant and CNV calling; long-read sequencing; RNA sequencing from disease-relevant tissues; functional minigene assays; segregation studies; and periodic reanalysis as variant interpretation resources evolve. These approaches have been shown to increase diagnostic yield in inherited retinal diseases [31, 32].

In this study, more than half of the patients with biallelic pathogenic *ABCA4* variants (16/27) were homozygous, and parental consanguinity was present in all homozygous patients. This result was similar to a previous Turkish study, which reported that 15 of 27 Turkish cases had homozygous variants [19]. Salles et al. reported only four homozygous cases among 40 Brazilian patients with biallelic *ABCA4* variants [29]. In a large Mexican cohort, the percentage of homozygous patients was 24% (51/211) [33]. The high percentage of homozygous patients in Turkish cohorts could be attributable to the higher rate of consanguineous marriages in Türkiye [34].

The most recurrent *ABCA4* variant in our cohort was c.5882G>A (p.Gly1961Glu), identified in 10 probands, and it has been reported as a recurrent variant in many studies, including the ProgStar study report-8 and the previous Turkish cohort by Sinim Kahraman et al. [19, 35]. However, c.52C>T (p.Arg18Trp), reported as a recurrent variant in the previous Turkish cohort, was not detected in our cohort [19]. Additionally, the second most recurrent variant in our cohort, c.4793C>A (p.Ala1598Asp), was not reported in previous Turkish cohorts [18, 19]. These differences may be attributable to the smaller size of Turkish cohorts or may indicate that different founder alleles play a role in populations from different geographic regions in Türkiye.

Our cohort included 27 patients with genetically confirmed STGD1; therefore, genotype–phenotype correlations were evaluated. For the severity of *ABCA4* variants, the classification criteria proposed by Lee et al. were used [24]. Additionally, the presence of biallelic LOF variants, including frameshift, nonsense, and splicing variants, was compared with the phenotypic characteristics of patients. The comparisons demonstrated that patients with biallelic severe variants and biallelic LOF variants had an earlier age of onset and more severe FAF findings, consistent with previous reports from larger cohorts [1, 7, 14, 21, 29, 19, 24, 35, 36]. These findings supported the correlation between the residual function of the ABCA4 protein and the clinical severity of the disease [37].

The p.Gly1961Glu variant is associated with milder disease. Patients with this variant generally have late-onset symptoms, and the FAF phenotype does not progress to type III, as reported in previous studies [14, 38]. This characteristic was also observed in our cohort, in eight patients who carried p.Gly1961Glu and presented after 10 years of age. FAF images of all patients were classified as type I or II. Only P20, who carried an additional frameshift variant affecting the second allele, had type II FAF findings at age 61 with a BCVA of 0.2. The phenotypic characteristics of these patients support the hypothesis that the p.Gly1961Glu variant is associated with milder disease.

Genetic test results led to reevaluation of the clinical diagnosis in five patients in the study cohort. Achromatopsia and CORD are two IRDs listed in the differential diagnosis of Stargardt disease [21, 39, 40] and may be misdiagnosed as phenocopies of Stargardt disease. Both Stargardt disease and achromatopsia may show optical gap appearance in OCT, as observed in our patients [39]. In severe cases of Stargardt disease, clinical findings did not distinguish CORD or RP from Stargardt disease [40]. Distinct electrophysiological features and the initial symptom of night vision loss can aid in the clinical diagnosis of enhanced S-cone syndrome [41]. Patient P48 was initially diagnosed with Stargardt disease due to the absence of ERG as part of the initial work-up; however, following genetic testing, the diagnosis was revised and subsequently confirmed as enhanced S-cone syndrome based on ERG findings. This highlights the importance of detailed phenotyping, including electrophysiological testing, for the accurate diagnosis of IRDs [42].

Late-onset Stargardt disease presents another diagnostic challenge in clinical practice, as it is often misdiagnosed as AMD and may not undergo genetic testing [13, 36]. In our cohort, three patients were clinically suspected of having late-onset Stargardt disease and underwent genetic testing. Genetic testing confirmed the clinical diagnosis in only one of them. In the Netherlands, the proportion of late-onset Stargardt disease was reported to be 33% among newly diagnosed and genetically confirmed patients [43]. The large difference between the two studies could be attributable to variations in study population size, referral rates for genetic testing, genetic testing methods, and population characteristics, including the mean age of the cohort [43, 44].

As far as we know, there have been no reports evaluating the process between clinical diagnosis and genetic testing among patients with Stargardt disease. In our study, the mean duration between referral by the ophthalmologist and the first visit with a medical geneticist was 13.8 days. A previous report by Morad et al. mentioned that the waiting time for first elective consultations was approximately 18 months in the Ocular Genetics Program at the Hospital for Sick Children in Toronto, and emphasized that reducing waiting times for consultation was a challenge [45]. In Morad’s report, the Ocular Genetics Program provided a more specialized multidisciplinary approach for all genetic eye diseases. However, our study showed that a multidisciplinary approach could be provided to patients in a shorter time in Türkiye. In our study, we also evaluated the duration between the first visit with a medical geneticist and the genetic counseling visit with test reports, which ranged from 4 to 13 months. This duration was considered reasonable since the clinic is a public institution with a very high patient volume, the medical geneticists are responsible for all genetic diseases, and there are no genetic counselors to share the workload in Türkiye. Additionally, the second visit includes not only the receipt of genetic test results but also the provision of genetic counseling. This comprehensive approach also contributed to the longer reported durations in this study. The multidisciplinary approach of ophthalmologists working with medical geneticists, along with genetic testing of all patients with a clinical diagnosis of Stargardt disease, resulted in confirmation of the clinical diagnosis in 54% of patients and reevaluation in 10%. Patients with a conclusive test result were given information about the prognosis of their disease based not only on their clinical findings but also on their genotype. Since ABCA4R is one of the hereditary eye diseases with relatively more reliable evidence of genotype–phenotype relationships, knowing the genotype may provide greater reassurance to both ophthalmologists and patients regarding disease prognosis [7, 14, 24].

This study has several limitations. First, the overall sample size, and particularly the number of cases in each genotype subgroup, was relatively small, limiting the strength of genotype–phenotype association analyses. Second, clinical investigations did not include electrophysiological tests for all patients, which could have contributed to clinical misdiagnosis. Third, missed copy number and deep-intronic variants in the *ABCA4* gene could account for cases with inconclusive genetic test results.

In conclusion, this study serves as a representative example demonstrating the contribution of genetic testing and a multidisciplinary approach to the management of IRDs in an ophthalmology clinic. Additionally, three novel *ABCA4* variants were identified in Turkish patients with Stargardt disease, and genotype–phenotype correlations were described. However, multicenter studies with larger sample sizes from Türkiye will be necessary to expand the *ABCA4* variant spectrum and provide more robust genotype–phenotype correlations.

## Supplementary Information





## Figures and Tables

**Table 1:** Genotype–phenotype characteristics of patients with conclusive genetic test results for STGD1.

Patient no	Genotype (*ABCA4* variants)	ACMG	Severity	AOA/AAE (years)	BCVA (OD/OS)	FAF Type
P1	c.6121G>A, p.Gly2041Ser	P	NA	17/27	0.1/0.1	II
P2*	c.5917delG, p.Val1973Ter	P	Severe	7/8	CF/CF	III
P3*	c.5917delG, p.Val1973Ter	P	Severe	15/25	0.05/0.05	II
P4	c.5882G>A, p.Gly1961Glu	P	Mild	29/32	0.3/0.3	I
P5	c.5018+2T>C	P	Severe	10/34	0.05/0.05	III
P6	c.4225A>G, p.Ile1409Val	LP	Moderate	11/37	0.1/0.1	II
P7	**c.466_467dupAT, p.Leu157SerfsTer2	P	NA	4/6	0.2/0.2	I
P8	c.3056C>T, p.Thr1019Met	LP	Severe	8/9	0.2/0.2	I
P9*	c.4793C>A, p.Ala1598Asp	LP	Severe	20/41	0.05/0.05	II
P10*	c.4793C>A, p.Ala1598Asp	LP	Severe	20/69	0.05/0.1	II
P11*	c.4793C>A, p.Ala1598Asp	LP	Severe	25/35	0.05/0.05	II
P12	c.1916A>G, p.Tyr639Cys	LP	NA	9/14	0.1/0.2	II
P13	c.5172G>A, p.Trp1724Ter	P	Severe	10/25	0.05/0.1	II
P14	**c.4540-1G>C	LP	NA	6/6	CF/0.05	I
P15	c.1807T>C, p.Tyr603His	LP	Moderate	7/12	0.1/0.1	I
P16	c.1834C>T, p.Gln612Ter	P	Severe	7/8	0.1/0.1	II
P17	A1:c.6568C>T, p.Gln2190Ter A2:c.5882G>A, p.Gly1961Glu	P/P	Mild NA	25/26	0.5/0.3	I
P18	A1:c.1832T>C, p.Leu611Pro A2:c.5882G>A, p.Gly1961Glu	P/P	Mild NA	18/27	0.1/0.1	II
P19	A1:c.4793C>A, p.Ala1598Asp A2:c.5882G>A, p.Gly1961Glu	P/P	Mild Severe	16/31	0.1/0.05	I
P20	A1:c.2069del, p.Gly690ValfsTer6 A2:c.5882G>A, p.Gly1961Glu	P/P	Mild Severe	14/61	0.2/0.2	II
P21	A1:c.1807T>C, p.Tyr603His A2:c.5714+5G>A	LP/LP	Moderate Moderate	18/20	0.2/0.2	I
P22	A1: c.1807T>C, p.Tyr603His A2:c.3383A>G, p.Asp1128Gly	LP/LP	Moderate Moderate	14/16	0.3/0.2	I
P23	A1:c.5018+2T>C A2:c.5882G>A, p.Gly1961Glu	P/P	Mild Severe	45/50	0.3/0.2	I
P24	A1:**c.878delG, p.Met293SerfsTer7 A2:c.5882G>A, p.Gly1961Glu	P/P	Mild NA	20/40	0.1/0.1	I
P25	A1: c.6568C>T, p.Gln2190Ter A2: c.4793C>A, p.Ala1598Asp	P/P	Severe NA	7/11	0.1/0.1	II
P26	A1:c.1622T> C, p.Leu541Pro A2: c.5882G>A, p.Gly1961Glu	P/P	Mild Severe	20/23	0.4/0.2	I
P27	A1: c.4793C>A, p.Ala1598Asp A2:c.5917delG, p.Val1973Ter	P/P	Severe Severe	9/10	0.2/0.2	I

*ABCA4* (NM_000350.3); A1, allele 1; A2, allele 2; ACMG, American College of Medical Genetics and Genomics; AEA, age at examination (years); AOA, age of ascertainment (years); BCVA, best-corrected visual acuity (Snellen); CF, counting fingers; FAF, fundus autofluorescence imaging; LP, likely pathogenic; P, pathogenic; NA, not available; OD, right eye; OS, left eye; VUS, variant of uncertain significance.

* Same family members (P2, P3; P9, P10, and P11).

** Novel variant.

**Table 2:** Genotype–phenotype characteristics of patients with conclusive genetic test results for other retinal dystrophies.

Patient no	Genotype	AOA/AAE (years)	Fundus	Diagnosis after genetic test
P38	*CNGA3*, A1: c. 1302G>A, p.Trp440Ter A2: c.1279C>T, p.Arg427Cys	1/8	Central macular atrophy without flecks FAF: Central and paracentral hypoautofluorescence OCT: Central outer retinal atrophy	Achromatopsia 2, AR
P39	*GUCY2D*, c.2513G>A, p.Arg838His	6/10	Subtle changes in the macula Central RPE atrophy OCT: optical gap appearance ERG: Decreased cone and rod responses, cone ERG was more severely affected than the rod ERG.	Cone-rod dystrophy 6, AD
P44	*PROM1*, c.1709dupA, p.Arg267Ter	10/28	Central retinal atrophy without flecks worse in OD with pigment clumps OCT: Central retinal and choroidal atrophy worse in OD	Cone-rod dystrophy 12, AR
P46	*CNGB3*, c.1006G>T, p.Glu336Ter	1/33	Central retinal atrophy, RPE changes, no fleck OCT: Central subfoveal outer retinal atrophy, cystic lesion	Achromatopsia 3, AR
P48	*NR2E3*, c.932G>A, p.Arg311Gln	7/8	Retinal atrophy areas with fibrotic scars, peripheral fleck like yellow-white deposits ERG: Absent rod response on scotopic ERG, the 30 Hz flicker response is of low amplitude and delayed	Enhanced S-cone syndrome, AR

*CNGA3* (NM_001298.3); *GUCY2D* (NM_000180.4); *PROM1* (NM_006017.2); *CNGB3* (NM_019098.5); *NR2E3* (NM_014249); A1, allele 1; A2, allele 2; ACMG, American College of Medical Genetics and Genomics; AD, autosomal dominant; AEA, age at examination (years); AOA, age of ascertainment (years); AR, autosomal recessive; ERG, electroretinography findings; FAF, fundus autofluorescence imaging; OCT, optical coherence tomography.

**Table 3:** Genotype–phenotype characteristics of patients with inconclusive genetic test results.

Patient no	Genotype	ACMG	AOA/AAE (years)	BCVA OD/OS	Fundus
P28*	*ABCA4*, A1: c.1239+1G>C A2: not detected	P	7/19	0.4/0.4	Central retinal atrophy without flecks
P29*	*ABCA4*, A1: c.1239+1G>C A2: not detected	P	18/32	0.1/0.1	Central retinal atrophy without flecks
P30	*ABCA4*, A1: c.1957C>T, p.Arg653Cys A2: not detected	P	62/64	1.0/0.8	Central retinal atrophy with flecks, foveal sparing
P31	*ABCA4*, A1: c.3808G>T, p.Glu1270Ter A2: not detected	P	34/36	0.3/0.1	Central retinal atrophy with flecks
P32	*ABCA4*, A1: Exon 9 deletion 144bp (CNV) A2: not detected	LP	4/12	0.1/0.1	Central retinal atrophy without flecks
P33	*ABCA4*, A1: c.5882G>A, p.Gly1961Glu A2: not detected	P	36/38	0.8/0.7	Paracentral macular atrophy without flecks and ring-like atrophy around vascular arcades
P34	*ABCA4*, A1: c.1917C>A, p.Tyr639Ter A2: not detected	P	16/19	0.5/0.5	Central retinal atrophy without flecks
P35	ABCA4, c.1654G>A, p.Val552Ile	VUS	4/8	0.3/0.4	Subtle foveal changes without flecks
P36	*ABCA4*, A1: c.455G>A, p.Arg152Gln A2: not detected	VUS	2/27	0.7/0.7	Central retinal atrophy without flecks
P37	*ABCA4*, A1: c.1654G>A, p.Val552Ile A2: not detected	VUS	58/62	0.7/0.05	Foveal sparing paracentral atrophy in OD and large central atrophy in OS
P40	No pathogenic variants detected	-	14/26	0.5/0.5	Central macular atrophy with peripheral light color oval flecks
P41	No pathogenic variants detected	-	8/10	0.05/0.1	Central retinal atrophy without flecks
P42	No pathogenic variants detected	-	7/29	0.2/0.2	Beaten bronze appearance of macula without flecks
P43	No pathogenic variants detected	-	40/42	0.2/1.0	Central retinal atrophy without flecks, hard exudates and CNVM in OD
P45	No pathogenic variants detected	-	9/15	0.4/0.4	Central retinal atrophy without flecks, foveal hyperpigmentation
P47	No pathogenic variants detected	-	3/7	0.1/0.16	Bull’s eye maculopathy without flecks
P49	No pathogenic variants detected	-	15/19	0.4/0.4	Bull’s eye maculopathy without flecks
P50	No pathogenic variants detected	-	6/32	0.05/0.05	Central retinal atrophy without flecks

*ABCA4* (NM_000350.3); A1, allele 1; A2, allele 2; ACMG, American College of Medical Genetics and Genomics; AEA, age at examination (years); AOA, age of ascertainment (years); BCVA, best-corrected visual acuity (Snellen); CNV; copy number variant; OD, right eye; OS, left eye; LP, likely pathogenic; P, pathogenic; VUS, variant of uncertain significance.

* Same family members (P28 and P29).
